# Supplementary figures and images for: Cryo-EM structure of the human somatostatin receptor 2 complex with its agonist somatostatin delineates the ligand-binding specificity
Source: eLife. 2022 Apr 21;11:e76823. doi: 10.7554/eLife.76823 (PMC9054131; doi:10.7554/eLife.76823)

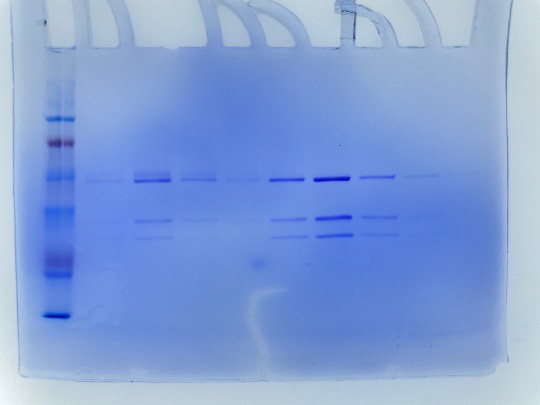

Supplement: Figure 1—figure supplement 1—source data 1. [file elife-76823-fig1-figsupp1-data1.zip › Figure1-Supple1-source1_uncroppedGel.jpg]

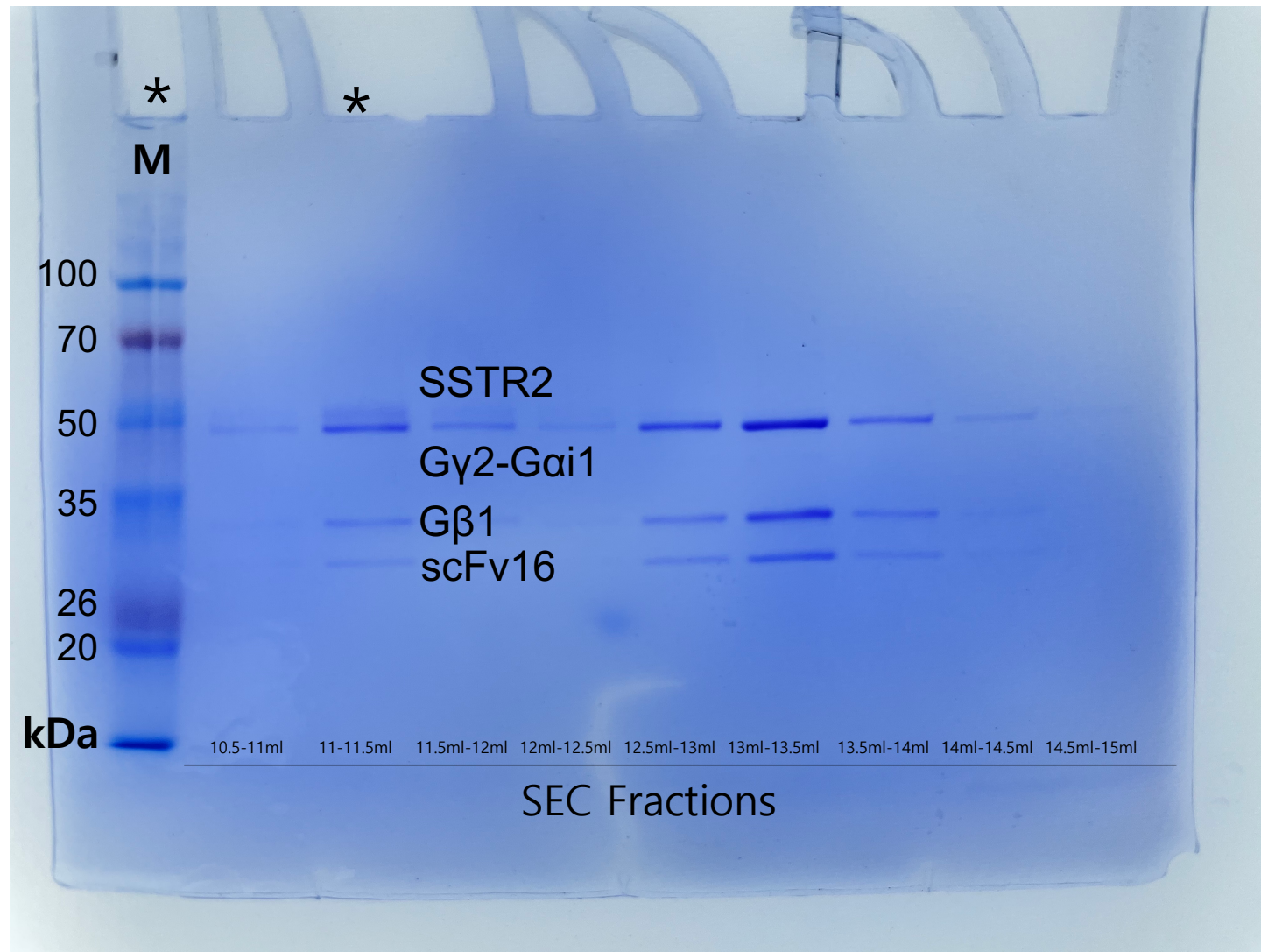

\*Lanes were used for Figure 1-figure Supplement 1

Supplement: Figure 1—figure supplement 1—source data 1. [file elife-76823-fig1-figsupp1-data1.zip › Figure1-Supple1-source1_withlabel.pdf]

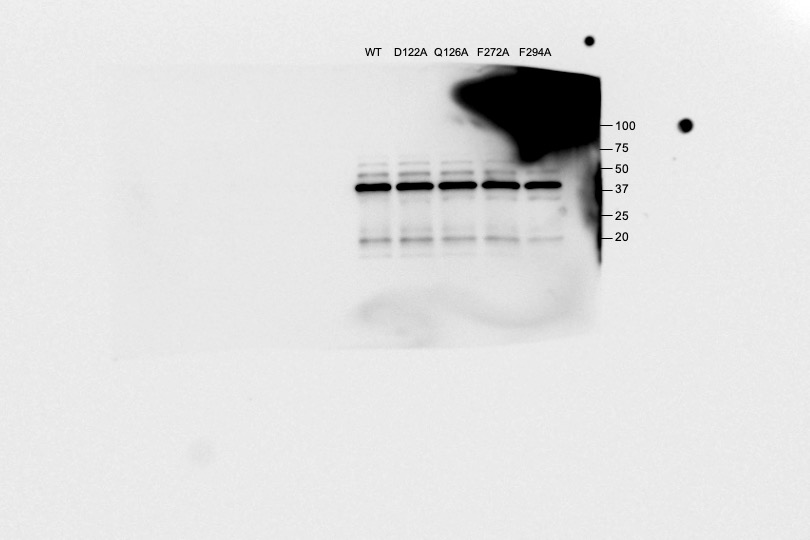

Supplement: Figure 2—figure supplement 1—source data 1. [file elife-76823-fig2-figsupp1-data1.zip › Figure2-figure_supplement1_source_data1_Actin.jpg]

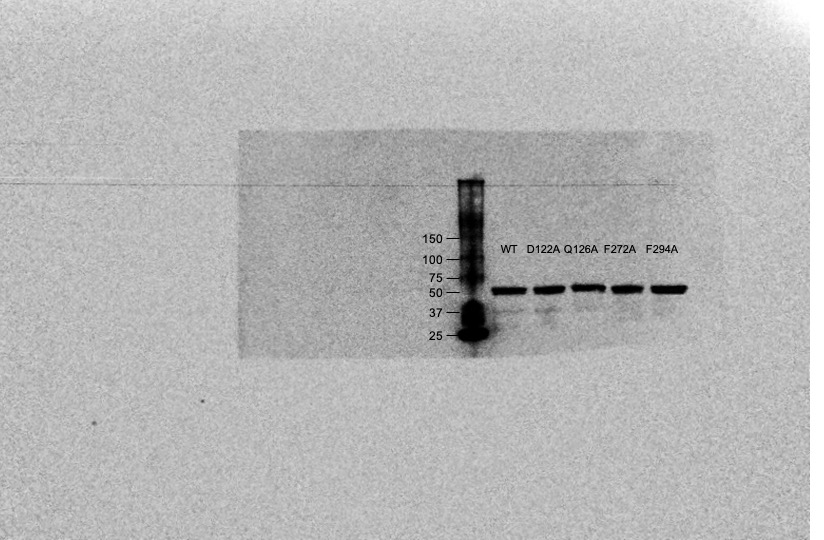

Supplement: Figure 2—figure supplement 1—source data 1. [file elife-76823-fig2-figsupp1-data1.zip › Figure2-figure_supplement1_source_data1_SSTR2.jpg]
